# Combination therapies for MPNSTs targeting RABL6A-RB1 signaling

**DOI:** 10.18632/oncotarget.27862

**Published:** 2021-01-05

**Authors:** Jordan L. Kohlmeyer, David J. Gordon, Munir R. Tanas, Rebecca D. Dodd, Varun Monga, Benjamin W. Darbro, Dawn E. Quelle

**Affiliations:** ^1^Molecular Medicine Graduate Program, University of Iowa, Iowa City, Iowa, USA; ^2^Department of Neuroscience and Pharmacology, University of Iowa, Iowa City, Iowa, USA; ^3^Department of Pediatrics, University of Iowa, Iowa City, Iowa, USA; ^4^Department of Pathology, University of Iowa, Iowa City, Iowa, USA; ^5^Department of Internal Medicine, University of Iowa, Iowa City, Iowa, USA; ^6^Holden Comprehensive Cancer Center, University of Iowa, Iowa City, Iowa, USA

**Keywords:** MPNST, RABL6A-RB1 signaling, CDK4/6 and MEK inhibitors, Ras, targeted cancer therapy

## Abstract

Precision medicine relies on a detailed molecular understanding of disease pathogenesis. Here, we consider urgently needed therapeutic options for malignant peripheral nerve sheath tumors (MPNSTs) based on emerging insights into druggable pathway alterations found to drive this deadly cancer. Recent observations demonstrate an essential role for an oncogenic GTPase, RABL6A, in promoting MPNST progression through hyperactivation of cyclin-dependent kinases (CDKs) and inactivation of the retinoblastoma (RB1) tumor suppressor. Monotherapies with CDK4/6 inhibitors have shown limited efficacy and durability in pre-clinical studies of MPNSTs and in clinical studies of other tumors. Therefore, we discuss the rationale and clinical benefits of inhibiting multiple RABL6A effectors, particularly CDK4/6 and MEK kinases, in targeted combination therapies suitable for MPNSTs and other Ras-driven malignancies.

## EDITORIAL COMMENTARY

Malignant peripheral nerve sheath tumors (MPNSTs) are aggressive, deadly soft tissue sarcomas that lack effective therapies [1–3]. These Schwann cell derived tumors arise spontaneously as well as in patients with the hereditary cancer predisposition syndrome, Neurofibromatosis Type I (NF1). The 5-year survival rate for patients with MPNSTs is only 20–35%, and MPNSTs are the leading cause of death in NF1 patients. In all contexts, loss of the Ras inhibitor, neurofibromin (encoded by the *NF1* gene), is a defining event in MPNST genesis [4]. Current treatment with traditional chemotherapy and radiation is highly toxic and does not reduce patient mortality, necessitating the development of more targeted treatments [5–9].

In most MPNSTs, cyclin-dependent kinases (CDKs) 2 and 4/6 are hyperactivated due to loss of their endogenous inhibitors, p16 and p27, or amplification of the cyclin and CDK genes [3]. This results in functional loss of the retinoblastoma (RB1) tumor suppressor, one of the most important guardians against cellular transformation and cancer development [10]. Because *RB1* remains genetically wild type in the majority of MPNSTs, its reactivation in tumors represents an exciting new approach for treating this disease.

**Figure 1:**
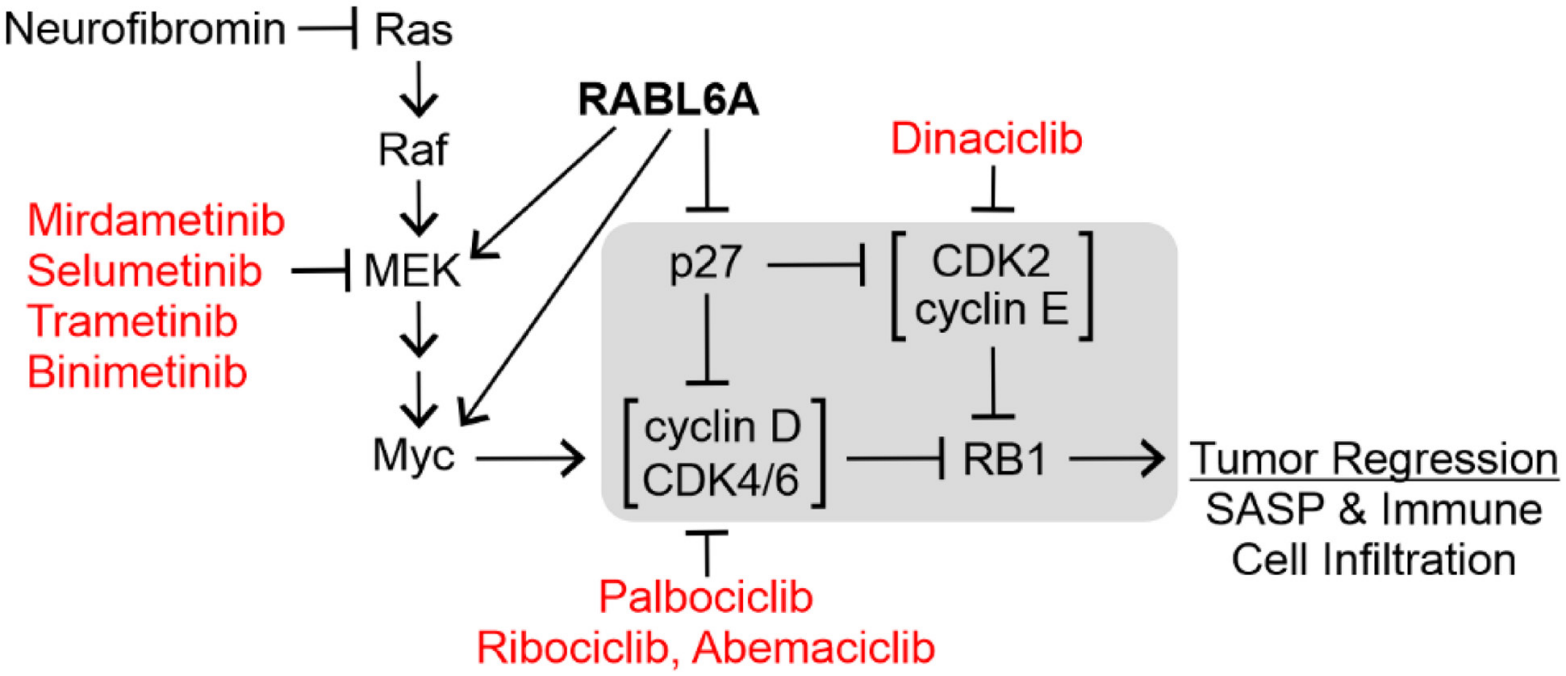
Pharmacologic targeting of the RABL6A-RB1 pathway in tumors. The central regulators of the RB1 tumor suppressor are highlighted in the gray box. Representative inhibitors of the indicated kinases are depicted in red. RABL6A inhibits RB1 by reducing the CDK inhibitor, p27, and activating MEK and Myc. Arrows, activating events; perpendicular bars, inhibitory events.

Recent studies of a novel RB1 regulator, named RABL6A, strengthens the rationale for RB1 targeted therapy in MPNST [11]. RABL6A is an oncogenic, RAB-like GTPase previously shown to inhibit RB1 signaling in pancreatic neuroendocrine tumors via downregulation of p27 [12]. Because p27 protein loss is a frequent event associated with worse MPNST patient survival [13], we explored RABL6A expression and function in those tumors. RABL6A protein was found to be robustly upregulated in patient MPNSTs relative to benign precursor lesions, with p27 expression patterns inverse to RABL6A levels [11]. Moreover, RABL6A knockdown studies showed it is required for MPNST cell viability and cell cycle progression. At the molecular level, RABL6A promotes MPNST proliferation and survival by decreasing p27 expression, increasing CDK4/6 activity and thereby inactivating RB1 (Figure 1). In addition, RABL6A has been shown to activate MEK [14, 15] and upregulate Myc mRNA [11, 16]. This can contribute to CDK-RB1 dysregulation because MEK and Myc are both hyperactivated in Ras-driven MPNSTs, and Myc is a transcriptional activator of *cyclin D, CDK4,* and *CDK6* genes [17–19]. Together, these observations reinforced the notion that MPNST growth would be strongly suppressed by reactivating RB1.

At present, the most effective way to reactivate RB1 in tumors is using drugs that selectively block CDK4/6 activity. CDK inhibitors were first introduced in the late 1990s as anti-cancer therapeutics; however, their lack of specificity for particular CDKs caused significant toxicity and undermined their usage in the clinic [3, 20–22]. Newer generation CDK inhibitors with greatly reduced toxicity include a group of related drugs with high specificity for CDK4/6, namely palbociclib, ribociclib, and abemaciclib (see Figure 1). These drugs are FDA-approved for the treatment of estrogen receptor-positive/HER2-negative metastatic breast cancer in combination with aromatase inhibitors [23, 24]. Excitingly, palbociclib and its relatives are showing great promise in clinical trials for other solid tumors, including another aggressive sarcoma, dedifferentiated liposarcoma (DDLPS) [25], which frequently harbors amplification of *CDK4* (NCT01209598, NCT02897375) [26].

With the above considerations in mind, we examined the efficacy of CDK4/6 inhibition in pre-clinical models of MPNST [11]. Reactivation of RB1 with palbociclib halted MPNST growth both *in vitro* and *in vivo* in orthotopic (sciatic nerve) mouse tumor models. The response to palbociclib was significantly reduced by RABL6A depletion in the tumor cells, suggesting that patient MPNSTs bearing elevated RABL6A may be more responsive to CDK4/6 inhibitor therapy. However, tumors in immunodeficient mice invariably acquired resistance to the monotherapy. This was not surprising since tumors in people and mouse models have been shown to employ many mechanisms to override the effects of CDK4/6 inhibitor monotherapy [24, 27, 28].

Upregulation of CDK2 is one of the most common mechanisms through which tumors overcome sustained CDK4/6 inhibition [27–30]. As depicted in Figure 1, CDK2 can compensate for the loss of CDK4/6 by phosphorylating and inactivating RB1. This, along with the high levels of CDK2 observed in many MPNST patient samples [11, 13, 31], prompted us to test combination therapy targeting both CDK4/6 and CDK2. Treatment with palbociclib plus the non-specific CDK2 inhibitor, dinaciclib, displayed greater antitumor activity in MPNST-bearing mice [11]. Unfortunately, dinaciclib was not well tolerated by the animals, reflecting the toxic effects of dinaciclib in patients that have limited its clinical use. This has steered ongoing investigations toward other RABL6A-RB1 targeted combination therapies.

Hyperactivation of Ras-MEK-ERK signaling is another way tumor cells acquire resistance to CDK4/6 inhibitor therapy as the pathway converges on RB1 [24, 32, 33]. This is illustrated by the fact activated MEK-ERK stimulates Myc, a transcription factor that directly increases the mRNA expression of CDK4, CDK6 and their regulatory partners, the D cyclins (Figure 1). Since MPNSTs are initiated by neurofibromin loss and enhanced Ras activity, they may have a higher likelihood or increased rate of resistance to CDK4/6 inhibition. Therefore, it stands to reason that agents targeting CDK4/6 in combination with clinically proven inhibitors of MEK may negate a key mechanism of resistance and effectively treat MPNSTs.

Indeed, the combination of CDK4/6 and MEK inhibitors has shown remarkable promise in pre-clinical and clinical studies of Ras-driven cancers, such as non-small cell lung cancer (NSCLC) and pancreatic adenocarcinomas [34–36]. The pre-clinical studies revealed a crucial role of drug-induced tumor cell senescence and the immune system in the anti-tumor response. The combination induces a senescence-associated secretory phenotype, called SASP, in which secreted cytokines promote the infiltration of immune cells (natural killer or CD8+ T cells) that cause tumor regression. The use of immune competent mouse tumor models is critical to observe that response and accurately evaluate the efficacy of dual therapies targeting CDK4/6 and MEK, such as palbociclib plus mirdametinib (Figure 1). This is possible for studies of MPNST given the availability of genetically engineered and CRISPR-based mouse models that develop *de novo* MPNSTs in the context of fully active immune systems [37–41]. To fully appreciate the complete role of the microenvironment in these studies, it will be important to evaluate therapies using multiple preclinical platforms, as murine background strain has a strong impact on the immune landscape of MPNSTs [42].

Clinical studies targeting CDK4/6 and MEK in solid tumors are highly encouraging. In a Phase 1 dose escalation study evaluating the combination of palbociclib with mirdametinib in patients with *RAS*-mutant solid tumors, promising progression free survival was reported. Of 25 patients, 11 were noted to be progression free for > 3 months with 6 patients displaying no disease progression for > 6 months. Moreover, 72% of patients achieved stable disease as their best response and 1 patient achieved partial response. This combination at maximum administered doses was deemed to be well tolerated [25]. A Phase 1b/2 trial evaluating the combination of ribociclib with binimetinib in *NRAS*-mutant melanoma recently reported their results from 41 patients treated on a 28-day schedule Phase 2 dose expansion cohort. The median duration of response was 10.3 months with a median time to progression and median progression free survival of 3.7 months. Slightly more than half of the patients (51.2%) achieved stable disease and 8 patients (19.5%) achieved partial response. The overall survival of the Phase 2 cohort was reported to be 11.3 months (NCT01781572). These are compelling results for *NRAS*-mutant melanoma patients since there are no targeted therapies currently approved. Other clinical studies evaluating similar combinations in *RAS*-mutant colorectal cancer (NCT03981614), *KRAS*-mutant NSCLC (NCT03170206), and in children and young adults with brain tumors (NCT03434262) are ongoing.

While there is good reason to be excited about the anti-tumor potential of therapies inhibiting both CDK4/6 and MEK, combination therapies targeting other RABL6A effectors may also have clinical efficacy in MPNSTs. That is because RABL6A upregulation is so prominent in this disease [11]. Moreover, RABL6A is a potent oncoprotein that controls many druggable cancer targets besides CDK4/6 and MEK, including Myc (Figure 1), PP2A-Akt-mTOR, and receptor tyrosine kinase pathways like VEGFR and EGFR [11, 12, 16]. As advances in drug development yield more specific targeted therapeutics, the future looks bright for the number of novel combination therapies that could be evaluated for rare malignancies, like MPNSTs, which currently lack effective therapy.
